# The Crabtree Effect Shapes the Saccharomyces cerevisiae Lag Phase during the Switch between Different Carbon Sources

**DOI:** 10.1128/mBio.01331-18

**Published:** 2018-10-30

**Authors:** Gemma Perez-Samper, Bram Cerulus, Abbas Jariani, Lieselotte Vermeersch, Nuria Barrajón Simancas, Markus M. M. Bisschops, Joost van den Brink, Daniel Solis-Escalante, Brigida Gallone, Dries De Maeyer, Elise van Bael, Tom Wenseleers, Jan Michiels, Kathleen Marchal, Pascale Daran-Lapujade, Kevin J. Verstrepen

**Affiliations:** a VIB – KU Leuven Center for Microbiology, Leuven, Belgium; b CMPG Laboratory of Genetics and Genomics, Department M2S, KU Leuven, Leuven, Belgium; c Department of Biotechnology, Delft University of Technology, Delft, The Netherlands; d Centre of Microbial and Plant Genetics, KU Leuven, Leuven, Belgium; e Laboratory of Socioecology and Social Evolution, Department of Biology, KU Leuven, Leuven, Belgium; f Department of Plant Biotechnology and Bioinformatics, Ghent University, Ghent, Belgium; Harvard Medical School

**Keywords:** Crabtree effect, lag phase, respiration, *Saccharomyces cerevisiae*, diauxic growth

## Abstract

When faced with environmental changes, microbes often enter a temporary growth arrest during which they reprogram the expression of specific genes to adapt to the new conditions. A prime example of such a lag phase occurs when microbes need to switch from glucose to other, less-preferred carbon sources. Despite its industrial relevance, the genetic network that determines the duration of the lag phase has not been studied in much detail. Here, we performed a genome-wide Bar-Seq screen to identify genetic determinants of the Saccharomyces cerevisiae glucose-to-galactose lag phase. The results show that genes involved in respiration, and specifically those encoding complexes III and IV of the electron transport chain, are needed for efficient growth resumption after the lag phase. Anaerobic growth experiments confirmed the importance of respiratory energy conversion in determining the lag phase duration. Moreover, overexpression of the central regulator of respiration, *HAP4*, leads to significantly shorter lag phases. Together, these results suggest that the glucose-induced repression of respiration, known as the Crabtree effect, is a major determinant of microbial fitness in fluctuating carbon environments.

## Abstract (precis)

The lag phase is arguably one of the prime characteristics of microbial growth. Longer lag phases result in lower competitive fitness in variable environments, and the duration of the lag phase is also important in many industrial processes where long lag phases lead to sluggish, less efficient fermentations. Despite the immense importance of the lag phase, surprisingly little is known about the exact molecular processes that determine its duration. Our study uses the molecular toolbox of S. cerevisiae combined with detailed growth experiments to reveal how the transition from fermentative to respirative metabolism is a key bottleneck for cells to overcome the lag phase. Together, our findings not only yield insight into the key molecular processes and genes that influence lag duration but also open routes to increase the efficiency of industrial fermentations and offer an experimental framework to study other types of lag behavior.

## INTRODUCTION

In their pioneering work in the early 1960s, Jacques Monod and François Jacob studied the growth kinetics of microbes in complex media (1). They found that populations of Escherichia coli growing in a mixture of glucose and a less-preferred carbon source such as galactose or lactose first consume the available glucose. When all the glucose is depleted from the medium, the microbes enter a temporary phase of slow growth, known as the lag phase, before resuming growth on the alternative carbon source (2). Along with follow-up studies of the E. coli Lac operon (3), this observation led to the revolutionary idea that the enzymatic activity in cells is regulated not only through direct feedback mechanisms between enzymes and their substrates but also through repression and activation of the genes encoding the respective enzymes (4). Specifically, it was found that glucose represses genes involved in the uptake and metabolism of alternative carbon sources, a process called glucose repression (5, 6).

Similar cases of diauxic growth were also described for many other microbes, including the common baker’s and brewer’s yeast Saccharomyces cerevisiae, which quickly established itself as a major model for the study of gene regulation in eukaryotes (7, 8). Glucose repression in S. cerevisiae is established through different pathways, most notably the protein kinase Snf1, the yeast homologue of the human AMPK, and the protein kinase A (PKA) pathways (reviewed in references 9 to 11). Interestingly, apart from repressing the metabolism of alternative carbon sources, glucose also affects the expression of genes related to other cellular functions such as respiration, gluconeogenesis, and general stress response mechanisms, explaining S. cerevisiae's preference for the fermentative dissimilation of glucose even in the presence of excess oxygen (9, 12, 13). The repression of respiration in cultures of this yeast growing in glucose-containing environments, also known as the Crabtree effect, has received special attention in the past, since other closely related yeast species actively use respiration to metabolize this sugar (13, 14). At first sight, the Crabtree effect is puzzling because the ATP yield per glucose unit is approximately one order of magnitude higher through respiration compared to fermentation (15). However, it is believed that fermentation allows higher fluxes and thus a higher rate of ATP production, and consequently faster growth (16). Moreover, the ethanol produced during fermentation can reduce the growth of competing microbes (17, 18). Interestingly, a similar repression of respiration in favor of high-flux fermentative metabolism also occurs in cancer cells, where it is known as the Warburg effect (19, 20).

Although the regulatory pathways involved in glucose repression have been studied in great detail, the factors that determine the length of the lag phase, characterizing the transition between glucose and glucose-repressed carbon source, remain largely unknown. The duration of the lag phase is obviously a key determinant of microbial fitness in variable environments, because a short lag time allows cells to resume growth more quickly than competitors with a longer lag. Moreover, the lag time is also important in industrial fermentations where microbes are often faced with a complex mixture of sugars in challenging environments (21). These complex media often result in long lag phases during which the fermentation speed is highly reduced (22). Such so-called “stuck” or “sluggish” fermentations cause great economic losses because they reduce the efficiency of a production plant and often also lead to inferior end products that contain undesirable fermentable sugars which make the product sweet and vulnerable to spoilage (9, 22).

Glucose-galactose mixtures have been extensively used as paradigm for diauxic growth in S. cerevisiae and the complex genetic switch enabling successive repression, derepression, and induction of the *GAL* genes has been well described (23). However, a very limited number of publications suggest that efficient expression of the *GAL* genes, which are directly involved in the uptake and metabolism of galactose (24), is not sufficient to enable a smooth transition between the two sugars (25, 26). To identify yet-unknown factors influencing the glucose-galactose shift, we use a large-scale barcode sequencing experiment (Bar-Seq) (27). By monitoring the lag phase of a collection of 4,887 mutants deleted for nonessential genes, we confirm that the *GAL* genes, which are directly involved in the uptake and metabolism of galactose (24), are crucial for escaping the lag phase. However, the genome-wide screen also reveals other genetic networks that are important determinants of the lag time. Specifically, networks involved in gene regulation and in respiration play key roles. Interestingly, overexpression of *HAP4*, a general regulator of respiration, drastically shortens the lag phase, suggesting that induction of respiration is a major rate-limiting step during the glucose-to-galactose lag phase. Moreover, we also show that the length of the lag phase differs greatly between a set of genetically diverse yeast strains, suggesting that genetic factors determine the speed at which cells can adapt to a new environment and resume growth (28, 29). Last, abolishing respiration by the nongenetic intervention of growth in the absence of oxygen also resulted in elongated lag phases of variable lengths. Together, these results shed light on the factors that determine microbial fitness in fluctuating environments and reveal that the glucose-to-galactose lag phase is not only determined by genes that are directly related to the uptake and metabolism of galactose but also by the capacity of each cell to express respiratory proteins and their energetic status. Moreover, the findings also open new routes for the improvement of industrial yeasts involved in the production of beer, wine, bread, and biofuels.

## RESULTS

### A genome-wide screen identifies deletion mutants with altered fitness in mixed sugar environments.

In the initial series of experiments, aimed at assessing which genes determine the duration of the lag phase when cells switch from glucose to less-preferred carbon sources, we used the Bar-Seq technology (30). Bar-Seq takes advantage of the DNA barcode carried by each mutant in the genome-wide S. cerevisiae deletion collection (31). Specifically, each deletion mutant has two different unique 20-bp barcode sequences, the so-called up (UP) and down (DN) barcodes, each flanked by primer sequences that are common across all mutants. These DNA tags make it possible to pool all 4,887 deletion mutants into one mixed culture and, by counting the proportion of the different barcodes using high-coverage sequencing, measure changes in the relative proportion of each of the 4,887 mutants in this pooled culture. When the pooled population of mutants is subjected to environmental changes, the relative fitness of each mutant can be determined by comparing the change in relative frequency between the initial and final samples of each experiment.

**FIG 1:**
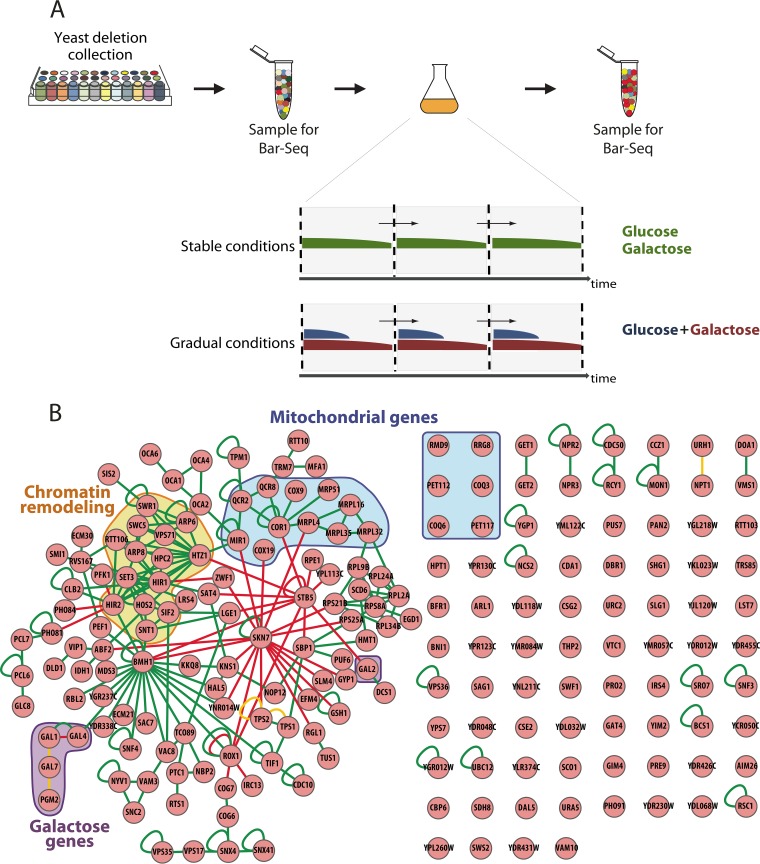
A genome-wide Bar-Seq screen reveals distinct cellular processes involved in adaptation to fluctuating carbon environments. (A) All the mutants from the yeast deletion collection were pooled in an aliquot and grown in two different regimes (stable and gradual conditions). For selection in stable conditions, the mutants were grown in media containing a single carbon source. For selection in gradual conditions, the pool of mutants was grown in low glucose (0.5%) supplemented with galactose (5%). At the start and after 3 rounds of selection, an initial sample and a final sample, respectively, were taken. These samples were used to determine enrichment of mutants through Bar-Seq. (B) Interaction network of the 200 genes that show the strongest depletion under gradual conditions. The edge colors correspond to protein-protein (green), methylation (yellow), and protein-DNA (red) interactions. The genes related to different cellular processes are highlighted: galactose metabolism (purple), chromatin remodeling (orange), and mitochondrial function and respiration (blue).

We set out to investigate the duration of the lag phase of the 4,887 available single-gene deletion mutants during a gradual switch from glucose to galactose. To obtain strong enrichment and depletion of mutants, the population was subjected to three rounds of this selection regime (Fig. 1A). In order to specifically assess the effect of the gene deletion on the lag phase duration, we aimed to minimize the effect of gene deletion on the growth speed in either glucose or galactose. To this end, we limited the number of doublings in glucose by reducing the glucose concentration to 0.5%. This limits the time the cells can grow on glucose but is sufficient to induce glucose repression. In addition, we allowed only 2 to 3 doublings of the population in galactose after the lag phase before the cells were transferred to a fresh mixture of glucose and galactose. Last, in order to estimate any remaining genetic effects related to growth speed on glucose or galactose alone, we also performed Bar-Seq analyses in rich media containing only glucose or only galactose (Fig. 1A).

### Bar-Seq experiments identify respiration as an important factor for high fitness in mixed sugar environments.

Following the Bar-Seq experiments, we calculated an enrichment score for each individual mutant under each condition (see Materials and Methods). The enrichment score in the mixed sugar conditions (glucose plus galactose) was corrected for the enrichment in pure glucose to account for the deletions that cause general slow growth. Based on this score, the 200 deletion mutants whose knockout genes resulted in longer lag phases were chosen for interaction network analysis. The interaction network analysis is based on known physical, genetic, and regulatory interactions and allows visualizing how the different genes that were picked up in the screen are connected by a common biological process or pathway (see Materials and Methods) (32).

Figure 1B shows the interaction network for the genes that, upon deletion, result in the strongest depletion in mixed sugar conditions. The network comprises several distinct groups of genes. As expected, one of these groups consists of the genes associated with galactose metabolism, thereby confirming their importance for the metabolic adaptation during the shift from glucose to galactose. One of the main hubs of the network is *SKN7*, a transcription factor which mediates oxidative stress response and is affected by the Ras/PKA pathway (33). Another central regulatory hub in the network is the gene *BMH1*, which encodes a protein involved in glucose repression (34) by regulating genes related to chromatin remodeling and histone deacetylation. A more surprising group of genes that stands out is the one containing genes encoding mitochondrial proteins, hereafter called mitochondrial genes. This group comprises mitochondrial ribosomal subunits as well as genes encoding subunits of different complexes in the electron transport chain. This is an unexpected but interesting result, since both glucose and galactose are known to sustain growth using fermentation (35), as opposed to glycerol or ethanol, which require respiration.

We also performed interaction network analysis on the genes that when deleted show the strongest enrichment score during a shift from glucose to galactose (see Fig. S1A in the supplemental material). A main group of genes in this network consists of cytoplasmic ribosomal genes. When analyzing the interaction network containing the genes for which deletion mutants exhibit the strongest depletion during growth in stable galactose conditions, we find genes related to vesicle transport, mitochondrial genes, and genes related to galactose metabolism (Fig. S1B). In the network corresponding to the genes with strongest enrichment during growth in stable galactose conditions, the largest group of genes encode cytoplasmic ribosomal subunits (Fig. S1C).

10.1128/mBio.01331-18.1 FIG S1 Interaction networks show the importance of different cellular processes in gradual and stable conditions. (A) Interaction networks of the 200 genes that when deleted show the strongest enrichment during the glucose-to-galactose shift. (B) Interaction networks of the genes that upon deletion show the strongest depletion when growing in galactose. (C) Interaction networks of the genes when deleted show the strongest enrichment when growing in galactose. In all networks, the edge colors correspond to protein-protein (green), methylation (yellow), and protein-DNA (red) interactions. Download FIG S1, PDF file, 0.6 MB. Copyright © 2018 Perez-Samper et al. 2018 Perez-Samper et al. This content is distributed under the terms of the Creative Commons Attribution 4.0 International license.

When comparing the interaction network corresponding to the genes with the strongest depletion during a glucose-to-galactose shift (Fig. 1B) and the corresponding network during growth in stable galactose (Fig. S1B), we see that, together with the *GAL* genes, mitochondrial genes are a common shared group in these two networks. This indicates that the expression of respiratory genes might play a role both in growth on galactose and in the metabolic adaptation during a glucose-to-galactose shift.

### A functional electron transport chain is required for a short lag time.

Our Bar-Seq screen identified several genes involved in respiration as important determinants of fitness in mixed sugar conditions. However, while Bar-Seq allows a quick and comprehensive screen to identify all genes that affect fitness in variable conditions, the results need to be approached with some caution. First, it is well-known that many mutants of the yeast deletion collection have accumulated mutations during its propagation and/or construction (36). Second, it is possible that mutants behave differently in a pool than in pure cultures. Third, the Bar-Seq data do not yield detailed insight into the exact change in lag phase and/or growth rate. We therefore aimed to confirm the Bar-Seq results by constructing new gene deletion mutants of the key genes identified through Bar-Seq. We focused on the set of respiratory genes and expanded the construction of deletion mutants to include more genes related to respiration. In total, we constructed 41 *de novo* deletion mutants, each with one selected respiratory gene deleted. These selected genes include 35 subunits and assembly factors of the electron transport chain as well as several subunits of the heme activator protein (HAP) complex, the coenzyme Q, and the cytochrome *c* complexes. Next, we examined the lag time of each of these mutants in separate cultures when growing in mixed sugar environments (0.5% glucose plus 5% galactose) as well as their growth rate in stable sugar conditions (growing only in galactose).

**FIG 2:**
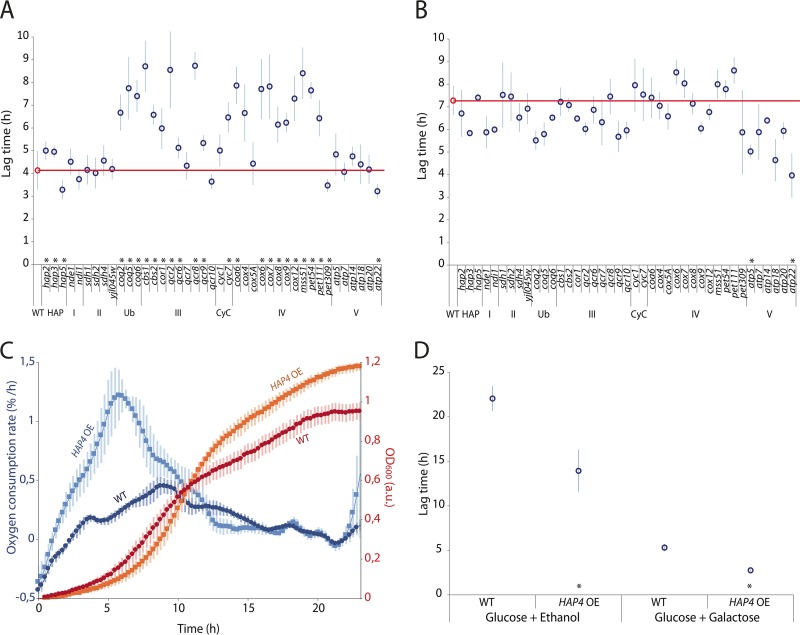
Respiratory activity affects the duration of the glucose-to-galactose lag phase. (A) Population lag times of respiratory deletion mutants growing on low glucose supplemented with galactose. The red circle and line indicate the lag time of the wild type. Error bars correspond to standard deviations of 3 or more biological replicates. *, *P* < 0.05 (two-tailed Student’s *t* test). (B) As in panel A but after the addition of antimycin A to the media. (C) Oxygen consumption rate and growth curve for the wild type and a strain with *HAP4* overexpression. Error bars correspond to standard deviations of 5 biological replicates. (D) Population lag time of the strain with *HAP4* overexpression when shifting from glucose to either ethanol or galactose. Error bars correspond to standard deviations of 3 or more biological replicates. *, *P* < 0.05 (two-tailed Student’s *t* test).

First, to assess the effects of the 41 respiratory gene deletions on the ability to adapt to a shift between sugars, we determined the length of their lag phase by performing population growth analysis in medium containing 0.5% glucose plus 5% galactose, which typically results in diauxic growth with a clear lag phase between the glucose and galactose growth. We find that the deletion of the investigated subunits generally resulted in a significantly longer lag time compared to the wild type, with cytochrome *c*, coenzyme Q, and the complexes III and IV of the electron transport chain being the ones affecting the lag time the most (Fig. 2A). The time to resume growth for a mutant in which a subunit of the above-mentioned complexes is deleted was about 2-fold longer than for the wild type (Fig. 2A). These results suggest that respiration and particularly the functionality of cytochrome *c* and complexes III and IV of the electron transport chain are critical for the cells to adapt to a new sugar in the environment.

To verify that these long lag times are indeed due to defects in respiration, we repeated these population growth experiments with antimycin A added to the growth medium. This compound binds to cytochrome *c* reductase, thereby inhibiting the electron flow through the electron transport chain and thus respiration (37). Addition of antimycin A prolonged the lag times up to 2.2-fold (Fig. 2B). Moreover, the addition of antimycin A to the media equalized the lag times of all mutants and the wild type, with the exception of the deletion mutants from complex V of the electron transport chain; their lag times became longer but not as long as for the wild type. Aside from the deletions of the subunits in complex V, the lag times of the deletion mutants are similar to the longest lag measured in a mutant without antimycin A added to the medium, suggesting that the changes in lag time in these mutants are indeed due to impaired respiration.

Second, we measured the growth rate of each of the mutants in stable sugar conditions (5% galactose; Fig. S2A). Deletion of 27 out of 41 respiration genes led to a significantly (*P* < 0.05) lower growth rate compared to the wild type. In order to confirm that these deletion mutants have altered growth rates because of impaired respiration, we also measured growth rates in the presence of antimycin A (Fig. S2B). All mutants grow at equally low growth rates in the presence of antimycin A, lower than the one that is observed for the slowest growing mutants in the absence of this compound. This indicates that the decreased growth rates shown by many respiratory deletion mutants are due to a lack of respiration and thus that cells rely at least partly on respiration to metabolize galactose. These results are in line with previous studies which report lower ethanol and higher biomass yields on galactose for wild-type yeast and hence a more respiratory metabolism of yeast cells when growing on galactose (38, 39).

10.1128/mBio.01331-18.2 FIG S2 Respiration is needed for efficient growth on galactose. (A) Growth rates in galactose of mutants in which specific genes involved in respiration were deleted. The red circle and line indicate the growth rate of the wild type. *, *P* < 0.05 (two-tailed Student’s *t* test). (B) As in panel A but the medium was supplemented with antimycin A. (C) Correlation between the growth rates of the respiratory deletion mutants when growing in medium containing galactose versus their lag times. The red circle indicates the wild type. Error bars in all panels correspond to standard deviations of 3 or more biological replicates. Download FIG S2, PDF file, 0.3 MB. Copyright © 2018 Perez-Samper et al. 2018 Perez-Samper et al. This content is distributed under the terms of the Creative Commons Attribution 4.0 International license.

In summary, our results show that impaired respiration leads to a reduced galactose growth rate and longer lag times during shifts to galactose. It is therefore possible that respiration is not specifically required during the lag phase but that any reduction of galactose growth rate, independent of its cause, will lead to a longer lag phase. Therefore, to investigate whether the necessity of respiration to overcome the lag phase is simply a consequence of its effect on the galactose growth rate or whether respiration serves a specific function during the lag phase, we correlated the lag times of all the respiratory mutants with their growth rates on galactose (Fig. S2C). Interestingly, the correlation is rather low (*R*^2^ = 0.267), so it is likely that the effect of respiration on the lag time cannot be exclusively explained by its effect on the growth rate in galactose.

### Overexpression of *HAP4*, a global regulator of respiratory genes, shortens the lag time.

While the previous results revealed that respiration and specifically complexes III and IV of the electron transport chain are crucial to efficiently escape the lag phase, this does not necessarily imply that the activation of respiration is a rate-limiting step that determines the length of the lag phase. To investigate whether increased respiratory activity results in a shortened lag phase, we overexpressed *HAP4*, the main transcription factor controlling the expression of respiration genes (40). To verify that the overexpression of this transcription factor causes higher respiratory activity in the cells, we compared the oxygen consumption rate of this strain with the wild-type strain, as well as their growth curves (Fig. 2C). This experiment was performed by growing the cells in glucose as the sole carbon source, a condition in which the cells should be primarily fermenting, and thus, little oxygen should be consumed. Our results show that the strain in which *HAP4* was overexpressed (*HAP4*-OE) had a higher oxygen consumption rate than the wild type did, especially during the initial exponential growth phase on glucose (Fig. 2C). Once we confirmed that the *HAP4*-OE strain had a higher respiratory activity than the wild type, we determined their lag time by performing population growth analysis during a glucose-to-galactose shift and as a control, also during a glucose-to-ethanol shift. As expected, we see that during a gradual shift from glucose to ethanol, the overexpression of *HAP4* causes a 40% reduction of the lag phase compared to wild-type cells (Fig. 2D). Interestingly, during the shift from glucose to galactose, the effect of *HAP4* overexpression on the duration of the lag phase is even more pronounced, reducing the lag time to about half of that of wild-type cells (Fig. 2D).

### Respiration and galactose genes are coinduced during the lag phase to galactose.

**FIG 3:**
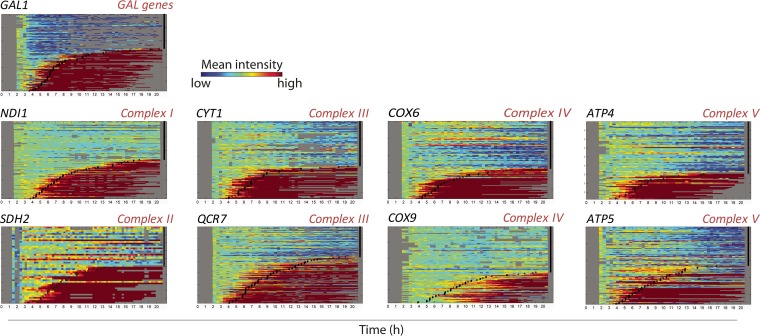
Genes involved in galactose metabolism and respiration are induced prior to the cells’ escape from the lag phase. The figure shows so-called kymographs of single cells during growth on galactose after 8 h of growth on glucose. We tracked microcolony growth of individual single cells expressing a specific fluorescently tagged protein (the corresponding gene name is specified in the panel). Every row represents the mean fluorescent intensity profile of the microcolony grown from a single cell. Black squares depict the moment at which each single cell starts growing on galactose. Note that the *GAL1* gene as well as genes involved in respiration (*NDI1*, *CYT1*, *COX6*, *ATP4*, *SDH2*, *QCR7*, *COX9*, and *ATP5*) are induced prior to each cell’s escape from the lag phase and that a large fraction of cells (typically 40% to 60% of the population) fail to induce these genes and also fail to resume growth on galactose.

After we confirmed the crucial role of genes involved in respiratory metabolism during the switch from glucose to galactose, we set out to assess the exact timing of the induction of these genes. Specifically, we measured the expression level of different representative subunits of each complex in the electron transport chain and of Gal1, a common reporter for *GAL* gene expression (41–43). These proteins were tagged using a fluorescent reporter, and the expression within single cells was tracked using fluorescence time-lapse microscopy. The results are represented in so-called kymographs, where each horizontal line represents a tracked cell and the color indicates the mean fluorescence in this cell as time progresses. In order to see the time of induction, the mean fluorescence level was normalized to the initial level, and the color scale was adjusted to visualize small changes from the initial level. The moment when a particular cell started growing is indicated by a black square (Fig. 3).

The initial observation is that not all the cells recover growth after the shift to galactose. Interestingly, only the cells inducing respiratory genes or genes involved in galactose metabolism are able to escape the lag phase and resume growth. The induction of these genes seems to precede escape from the lag, especially when taking into account the maturation time of the fluorescent reporter and its detection limit.

### Respiration affects lag phase by maintaining the cellular energy status and enabling translation of the Gal enzymes.

**FIG 4:**
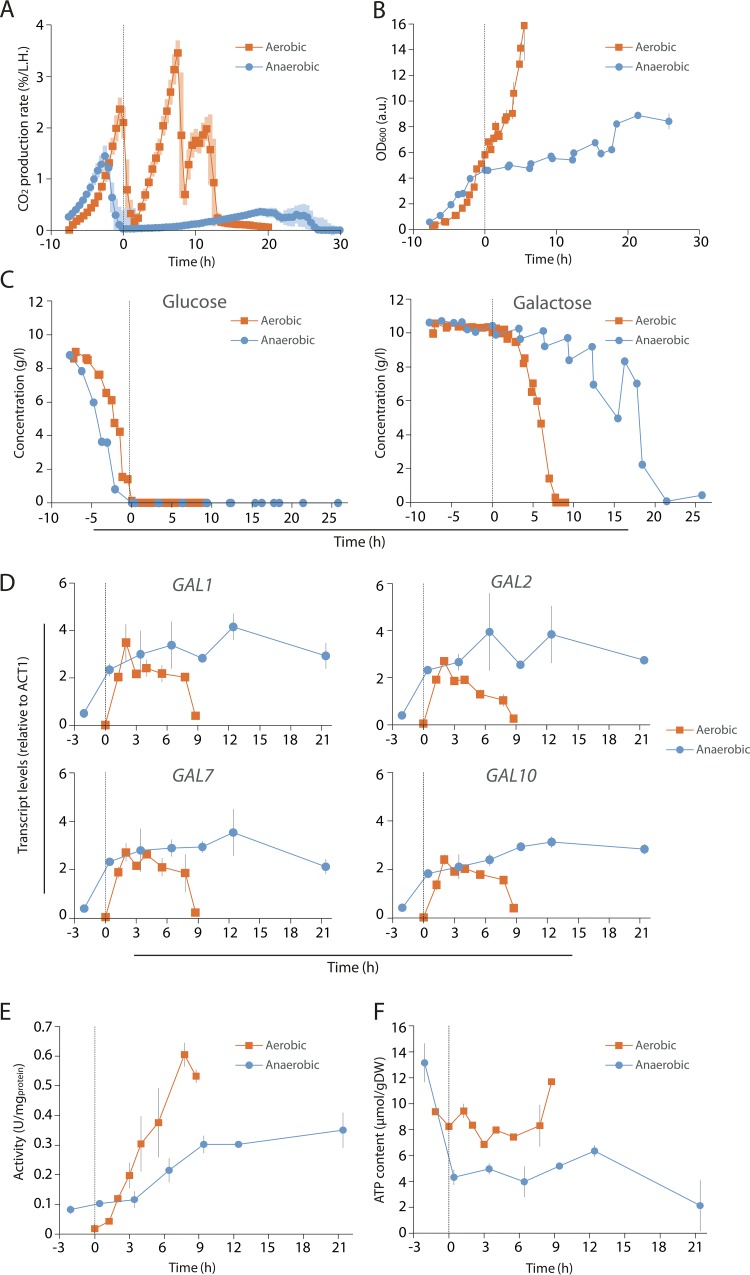
Respiration affects lag phase by maintaining the cellular energy status and enabling translation of the Gal enzymes. (A) Volumetric CO_2_ production rate profiles of bioreactor batch cultures of S. cerevisiae CEN.PK113-7D grown on glucose-galactose mixtures in aerobic and anaerobic conditions. The shadows around the curves correspond to the standard deviation of 6 (aerobic) and 7 (anaerobic) biological replicates. (B) Optical density over time for aerobic and anaerobic growth. Error bars correspond to standard deviations of 2 biological replicates. Optical densities corresponded well with cell weight (dry weight) concentrations. (C) Glucose and galactose concentrations. (D) Relative expression of *GAL* genes during aerobic and anaerobic growth. Error bars correspond to standard deviations of 2 biological replicates. (E) Galactokinase activity during aerobic and anaerobic growth on glucose-galactose mixtures. Error bars correspond to standard deviations of 2 biological replicates. (F) Intracellular ATP pools. Error bars correspond to standard deviations of 2 biological replicates. In all panels, aerobic conditions are depicted in orange and anaerobic conditions in blue. Moreover, the time of glucose depletion, corresponding to the shift to galactose consumption is set as time zero and depicted as a dashed line.

To explore the mechanisms underlying this correlation between respiration and lag phase, the glucose-galactose transition of a prototrophic S. cerevisiae strain was monitored during growth in tightly controlled bioreactors. For this physiological study, we chose a laboratory strain from the CEN.PK background, intensively used for fundamental and applied biotechnology. Also in this strain background, similar to the AN63 strain, deletion of a complex III subunit led to a prolonged lag phase during the diauxic shift from glucose to galactose (Fig. S3A). The fact that two different S. cerevisiae strains show similar behavior strongly supports that the observed correlation between the length of lag phase and respiration is universal for S. cerevisiae strains. In addition to the use of respiratory-deficient mutant strains or respiratory chain inhibitors (e.g., antimycin A), we employed an alternative approach to investigate the importance of respiration during the switch from glucose to galactose. This is based on the unique property of S. cerevisiae to rapidly proliferate in the absence of oxygen with minimal supplements (44). Oxygen deprivation enables yeast to tune the energy supply, as it disables cells from generating ATP via oxidative phosphorylation despite the presence of a fully functional respiratory chain. In line with results of other non-respiring cultures, cultivation in the absence of oxygen resulted in extremely long lag phases and long galactose consumption phases (Fig. 4A). Furthermore, large variations in the duration of these phases among replicate cultures were observed for the anaerobic cultures. Remarkably, while the increase in OD_600_ in these cultures was detected only 15 (±3) hours after glucose exhaustion (Fig. 4B), galactose utilization was resumed at a low rate shortly (2 to 4 h) after glucose exhaustion (Fig. 4C). These results show that respiration is not required to activate the GAL system, which is confirmed by the rapid and strong induction of the *GAL* genes upon glucose depletion (Fig. 4D). Conversely, in the absence of oxygen, cells display slower and lower expression of galactokinase, the first protein in the galactose utilization pathway (Fig. 4E). Combined with the low ATP content of non-respiring cultures compared to respiring cultures (Fig. 4F), these results suggest that respiring cells can more efficiently conserve energy from galactose, thereby enabling the actual synthesis of required proteins. A stronger reduction in culture viability under nonrespiring conditions might explain an increased lag phase at the whole-population level. However, fractions of dead cells in both oxygenated and anaerobic cultures remained below 4% (Fig. S3B). Respiration is thus not essential to maintain viability after glucose depletion.

10.1128/mBio.01331-18.3 FIG S3 (A) CO_2_ production of the CEN.PK prototrophic strain IMK242 carrying a *RIP1* deletion (van den Brink et al. [25]) when cultivated in aerobic bioreactors (as described in Materials and Methods) and compared to its isogenic reference strain S. cerevisiae CEN.PK113-7D during aerobic and anaerobic growth on glucose-galactose mixtures. For each culture condition, at least four independent culture replicates are shown. (B) The percentage of dead cells (PI positive) was used to assess the viability of aerobic and anaerobic batch cultures. Error bars correspond to standard deviations of 2 biological replicates. The time of glucose depletion, corresponding to the shift to galactose consumption, is set as time zero and depicted as a dashed line. Download FIG S3, PDF file, 0.3 MB. Copyright © 2018 Perez-Samper et al. 2018 Perez-Samper et al. This content is distributed under the terms of the Creative Commons Attribution 4.0 International license.

### Natural variation in the lag time of different strains is correlated with the level of respiration during glucose growth.

**FIG 5:**
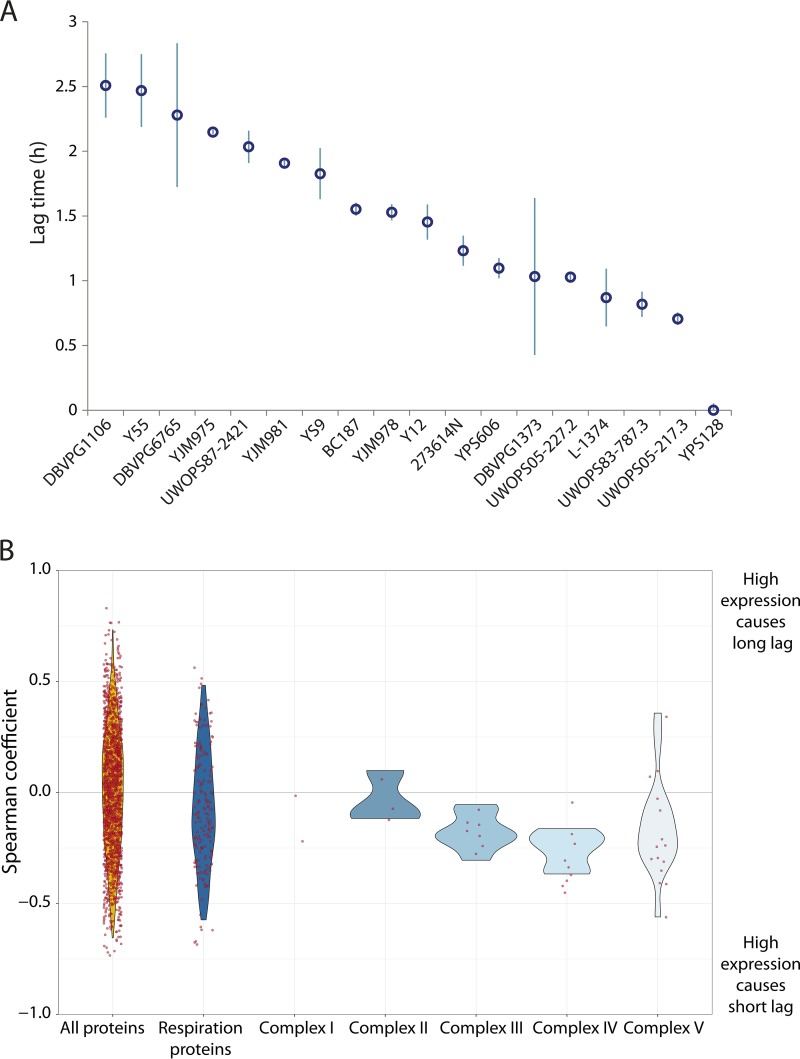
The glucose-to-galactose lag times of different natural S. cerevisiae strains correlate with their expression of respiratory proteins in medium containing glucose. (A) Population lag times of 18 different S. cerevisiae strains during a gradual shift from glucose to galactose. Error bars correspond to standard deviations of 3 or more biological replicates. (B) Violin plots of the Spearman correlation coefficients between the protein expression obtained by Skelly et al. (45) of 18 S. cerevisiae natural strains and their lag times when shifting from glucose to galactose. Red dots indicate correlation coefficient of individual proteins.

Next, we investigated whether natural variation in respiratory activity during growth on glucose might explain some of the variation in lag duration observed between genetically different strains of S. cerevisiae. For this analysis, we used data obtained by Skelly and coworkers, who conducted comprehensive proteomics and transcriptomics analyses to show that different natural S. cerevisiae strains contain significantly different levels of proteins that can be linked to fermentation and respiration when grown in chemostats in glucose-rich conditions (45). We measured the lag times of 18 of these natural S. cerevisiae strains when the strains were shifted from glucose to galactose (Fig. 5A). These results reveal considerable differences in the lag duration of these strains, with lag times differing from almost 0 to 2.5 h.

Next, we calculated the Spearman correlation coefficient between the lag duration of the different S. cerevisiae strains and their reported protein quantities (Fig. 5B). When correlating all the proteins investigated by Skelly et al. (45) with the lag times we measured, the Spearman coefficients follow a normal distribution, meaning that there is no general bias of the data toward positive or negative correlations. However, when we examine only the proteins related to respiration, as determined by Steinmetz et al. (46), the distributions of correlation coefficients show a slight bias for negative values, indicating that low levels of proteins involved in respiration are correlated with long lag durations. Specifically, when we calculate the correlations between lag duration and the levels of proteins linked to the different electron transport chain complexes, the distribution of coefficients clearly shifts to the negative side of the correlation, meaning that high expression of these proteins is predictive of a short lag time. This is particularly true for complexes III and IV, whose correlation coefficients are at the lower side of the spectrum. These findings are in agreement with our results obtained by deleting 41 different respiration genes, which point to complexes III and IV of the electron transport chain as the components with more influence on the speed of growth adaptation (Fig. 2).

In summary, these data reveal that natural variation in the expression of respiratory proteins of the electron transport chain correlates with the length of the lag phase, making the strains with higher expression of these proteins more likely to adapt quickly to nutritional changes in the environment.

## DISCUSSION

In 1949, Jacques Monod characterized the different phases of cell growth when microbes adapt their metabolism to a fluctuating nutritional environment (2). He defined the lag phase as the period of time needed for the cells to reprogram their metabolism and express the enzymes required for growth in the secondary carbohydrate. Monod already understood the importance of the inhibitory effect of the first consumed carbon source, suggesting the existence of glucose repression. Later studies of the *lac* operon in E. coli confirmed that glucose represses transcription of the genes involved in the uptake and metabolism of lactose (3). Soon, other examples followed, including the glucose-induced repression of the galactose (*GAL*) genes and respiratory genes in S. cerevisiae. This repression of respiration by glucose was first described by Herbert Crabtree in 1928 and was therefore called the “Crabtree effect” (47). It was later shown that several other yeasts show a similar tendency toward fermentation and thus are also Crabtree positive (14, 48). In this way, the regulatory network underlying glucose-induced repression of genes involved in the uptake and metabolism of alternative carbohydrates, e.g., galactose, became one of the most studied models for gene regulation (reviewed in reference 9). Nowadays, the exact dynamics and response of the galactose system are well characterized, with studies determining the importance of the feedback loops in the *GAL* system (49) or studies showing the specific roles of the different GAL proteins (50, 51). However, despite much progress in the characterization of the network underlying glucose repression, few studies have investigated how this signaling relates to the duration of lag phase and which other processes might be involved in the adaptation to alternative carbon sources (25, 41, 42, 52).

Our work confirms that the lag phase associated with glucose depletion is defined by the derepression of genes involved in the uptake and metabolism of alternative carbon sources. Although it has been shown that *MIG1* and *GAL80* play a crucial role in determining the length of the lag phase (39), our genome-wide Bar-Seq screen revealed that many more genes are important for cells to efficiently escape the lag phase (Fig. 1A). These genes include genes involved in the glucose repression pathway as well as key genes involved in transcriptional regulation and chromatin remodeling. Previous studies demonstrate that at low glucose concentrations, the protein kinase Snf1 is partially activated and that there is a certain degree of glucose derepression (53), but our results suggest that glucose repression is sufficiently strong to be the key determinant of the lag duration. Perhaps more surprisingly, our results also reveal that one particular cellular activity that is repressed by glucose, namely respiration, is a key limiting factor for the efficiency with which S. cerevisiae cells can switch between carbon sources. Whole-genome screenings, population growth analysis, and single-cell fluorescence data all showed that efficient escape from the lag phase requires derepression of genes involved in respiration.

Specifically, our results show that complexes III and IV of the electron transport chain are the key elements in the adaptation to galactose. In addition, we also show that the variation in lag duration between different feral S. cerevisiae strains correlates with the expression of complexes III and IV of the electron transport chain, suggesting that a higher respiratory activity in glucose may be correlated with a faster progression through the lag phase when glucose is depleted. It has been reported that these two complexes of the electron transport chain of S. cerevisiae cluster together in the inner membrane of mitochondria forming a supercomplex (54). The deletion of subunits of these complexes leads to the disassembly of the supercomplex, rather than rendering the respective complexes completely nonfunctional (55). Considering these observations and our results, we hypothesize that the length of the lag phase might be strongly influenced by the stability of this supercomplex. If complexes III and IV of the electron transport chain cannot aggregate and form the supercomplex, the transfer of electrons throughout the whole electron transport chain cannot be completed. We also demonstrate that cells already express the genes encoding the proteins of the electron transport chain before they start growing in the alternative carbon source (Fig. 3). These observations are in line with the extremely long lag phases observed in cultures grown in the absence of oxygen (Fig. 4), which illustrates the importance of oxidative phosphorylation during diauxic shifts.

Interestingly, defective or repressed respiration has also been reported as the cause of numerous human diseases (56). Several studies make use of S. cerevisiae to show that conditions like diabetes, obesity, or cardiovascular disorders are caused by a defect in mitochondrial function, and more precisely, by insufficient respiration (19, 56). One of the most studied diseases in which respiration plays a crucial role is cancer. In 1927, Otto Warburg described how cancer cells with deficient respiration switch their metabolism from respiration to fermentation (57). This “Warburg effect” was later shown to be crucial for the survival of oncocytes, epithelial carcinogenic cells present in kidney, salivary glands, or thyroid cancers (58). In fact, it is believed that these types of tumors shift from respiration to fermentation due to a mitochondrial dysfunction, and more specifically, due to the destabilization of the supercomplex of the electron transport chain. Thus, in both yeast and cancer cells, growth is intrinsically linked to mitochondrial respiration-related genes.

Apart from revealing the broad network of genes that control the lag phase and the central role of respiration, our results also have practical implications. Many industrial fermentation processes, including the production of beer, wine, and bulk chemicals like bioethanol, use a mixture of glucose and less-preferred carbon sources and are performed under anaerobic environments. This implies that the microbes undergo a lag phase as they switch from glucose to the nonpreferred carbon sources. In some cases, the lag phase can be excessive, and such so-called “sluggish” or “stuck” fermentations can cause great economic losses (9, 22). Our results show that the duration of the lag phase in glucose-galactose mixtures is strain dependent. Our results also suggest, in line with earlier reports, that supplying a small amount of oxygen at the time of the switch to activate respiration might enable cells to escape the lag phase and to start metabolizing the alternative carbon sources. Alternatively, the present study is a starting point for metabolic engineering strategies to improve yeast efficiency in mixed-substrate fermentations.

An intriguing observation is that a large fraction of cells in a population are unable to shift from glucose to galactose (Fig. 3; see also Fig. S3 in the supplemental material). While it has been well documented that substrate transition leads to stochastic gene regulation (3, 28, 59, 60), the present study suggests that population heterogeneity during substrate transition does not result from stochastic variations in transcription of the *GAL* genes but rather hinges on the respiratory activity and metabolic state of the cells (Fig. 4 and Fig. S3). During cultivation in an anaerobic environment, induction of the *GAL* genes at the transcriptional level was not coupled to efficient synthesis of the corresponding proteins. Furthermore, respiratory activity, which is by far the most efficient cellular process in terms of energy conservation, has a strong impact on the duration of the lag phase during glucose-galactose transition. Considering that protein synthesis is by far the most ATP-intensive biosynthetic process (61), it is tempting to speculate that the ability of individual S. cerevisiae cells to initiate galactose utilization is strongly influenced by their energy status. However, further research is needed to investigate whether this is really the case.

## MATERIALS AND METHODS

### Yeast strains and media used.

#### (i) Creation of the yeast deletion collection pool.

All 96-well plates from the yeast deletion collection were thawed, and 3 µl of each well was transferred to a new 96-well plate with 150 µl of YP (20 g/liter bacterial peptone, 10 g/liter yeast extract) with 20 g/liter of glucose and containing 200 µg/ml of G-418 disulfate. The cultures were grown until all reached an OD_600_ higher than 1. To create the pool of mutants, 50 µl from each well of each 96-well plate was added into a beaker. After proper mixing, 1 ml of the pool of mutants was aliquoted and frozen.

#### (ii) Construction of respiration deletion mutants.

All deletion mutants were made using the standard transformation protocol with the S. cerevisiae strain AN63 (28) (BY4742 *SAL1*^+^ *MAL*^+^ *MAT***a**) as the background strain. For each deletion mutant, a PCR product was obtained after amplifying the NatR marker from the plasmid pYM-N19 with specific primers (see Data Set S1 in the supplemental material). The primers used to verify the deletion of each specific gene are listed in Data Set S1.

10.1128/mBio.01331-18.4 DATA SET S1 Strains and primers used in this study. Download Data Set S1, XLSX file, 0.02 MB. Copyright © 2018 Perez-Samper et al. 2018 Perez-Samper et al. This content is distributed under the terms of the Creative Commons Attribution 4.0 International license.

#### (iii) Construction of fluorescently tagged mutants.

The standard transformation protocol was also used to fluorescently tag genes from the electron transport chain, as well as the *GAL1* gene with yECitrine. The AN63 strain (28) was used as the background strain. For each transformation, a PCR product was obtained from the plasmid pKT140 with specific primers (Data Set S1). The primers used to verify the proper insertion of the fluorescent marker for each gene are listed in Data Set S1.

#### (iv) Construction of *HAP4*-OE strain.

The *HAP4*-OE strain was made using standard transformation protocol with the S. cerevisiae strain AN63 (28) (BY4742 *SAL1*^+^ *MAL*^+^ *MAT***a**) as the background strain. The PCR product was obtained after amplifying the NatR marker from the plasmid pYM-N19 with specific primers (Data Set S1). The primers used to verify the overexpression of the *HAP4* gene are listed in Data Set S1.

### Bar-Seq experiments.

#### (i) Performance of the experiments.

One aliquot from the yeast deletion collection pool was thawed and recovered overnight in 50 ml YPD at 30°C shaking at 200 rpm. The next day, a sample of the initial pool of mutants was taken by transferring 45 ml of the culture to a Falcon tube and spinning it at 3,000 rpm for 3 min. The pellet was resuspended in 25% glycerol and frozen at −80°C. From the remaining 5 ml of culture, the OD_600_ was measured using an automated cell counter (Bio-Rad), and the inoculum for cell growth in the next round was calculated so that after 6 to 8 doublings, the final OD_600_ of the culture would be 0.1. For each of the two biological replicates, the calculated volume was transferred to an Eppendorf tube and washed three times with the same medium used for cell growth in the following rounds. The washed cells were inoculated into 200 ml of media containing either YP plus 5% glucose or YP plus 5% galactose. After inoculation, OD_600_ measurements were taken regularly. Once a culture reached an OD_600_ of 0.1, 50 ml of culture was used to take a sample from that specific condition. At the same time, the inoculum for the next round was calculated and washed with fresh media containing YP plus 5% of the corresponding sugar. The flask for the next round was inoculated with the washed cells. This procedure was followed until 3 rounds of growth were completed.

For the Bar-Seq experiment with gradual conditions, the same protocol was followed replacing the carbon sources used by YP plus 0.5% glucose plus 5% galactose.

#### (ii) Sample preparation and data analysis.

The first sample and the last sample of each growth condition from the Bar-Seq experiments were thawed, and genomic DNA was extracted using the standard zymolyase protocol. The UP and DN barcodes of those samples were amplified with two different PCRs and primer sets (Data Set S1) using *Ex Taq* polymerase (TaKaRa) and its standard cycling conditions, with 55°C as the annealing temperature and 30-s elongation time. The PCR product was purified using the QIAquick kit (Qiagen). DNA concentration was measured with Qubit (ThermoFisher). After equalizing the concentration of all PCR products to 5 ng/µl, the samples were sent for Illumina MiSeq sequencing.

Sequencing data were analyzed by blasting the Illumina reads to the known barcode sequences of the deletion mutants retrieved from http://www-sequence.stanford.edu/group/yeast_deletion_project/. Three gaps and two mismatches were allowed. In each sample, the frequency of each deletion mutant was calculated by dividing its counts by the total counts for that sample.

The enrichment of each particular deletion mutant in the samples of all Bar-Seq experiments was calculated. This was done by taking the proportions of the mutant in the initial and final samples into account, as well as the increase in cell number during the experiment and the total time it lasted.

However, some deletion mutants disappeared throughout the experiment, i.e., they were present in the initial sample but not in the final sample. For these deletion mutants, the enrichment was estimated by considering their proportion in the initial sample and evaluating the maximum growth rate at which these deletion mutants should have grown in order to not be present in the final sample. The normalized enrichment of each mutant was calculated by dividing its enrichment on the alternative sugar by the one from the glucose condition. In this way, we accounted for the effect of the secondary sugar in the media. Next, we considered the normalized enrichment of each deletion mutant to obtain separate ranking lists for UP and DN barcodes for each replicate and each growth condition, resulting in eight different ranking lists. In order to determine the performance of each mutant in a certain growth condition, we averaged the ranking positions of each mutant for two replicates and for the UP and DN barcodes, resulting in a single ranking list of mutants per growth condition. Next, we considered the 200 genes in the extremes of the normalized enrichment distributions for each growth condition. To determine which 200 mutants had the strongest normalized enrichment, we build a strongly enriched distribution. For that, all the mutants for which the enrichment could be calculated as well as the mutants that disappeared when growing on glucose alone (for which the enrichment was estimated) were taken into account. As for the weakly enriched, a distribution was made by considering all the mutants for which the enrichment was calculated and the mutants that disappeared when growing on the secondary sugar (for which the enrichment was estimated). Once we defined both strongly and weakly enriched distributions, we ordered the mutants in each distribution considering their ranking position in the ranking lists described above. With these ordered distributions, the 200 mutants with strongest and weakest normalized enrichments were selected for further analysis.

### Network analysis.

Interaction networks were built using Cytoscape software. The background network used for all known interactions was the one used in PheNetic (32). The STRING interaction database was used to extract the protein-protein interactions (62), YEASTRACT was used for the protein-DNA interactions (63), and the metabolic networks were retrieved from the KEGG database (64). After uploading to Cytoscape the list of genes corresponding to the deletion mutants that we wish to analyze together with their ranking score, a filter was applied to the background network so that the 200 genes with the strongest or weakest scores were selected. These selected genes were used to create an interaction network by applying force directed layout. Cytoscape was exclusively used as a visualization tool.

### Population growth lag time measurements.

All population growth measurements were performed using Bioscreen C machines (Growth Curves USA). For the gradual shift experiments, cells were inoculated in a 96-well plate containing YP plus 5% glucose (high-glucose [HG]) medium and serially diluted for growth overnight at 30°C shaking at 900 rpm. Cultures at an OD_600_ of 0.1 were transferred to a new plate and serially diluted in fresh HG medium for growth overnight. The next day, cultures at an OD_600_ of 0.1 were washed three times into YP plus 0.5% glucose (low glucose [LG]) plus the appropriate carbohydrate source, with or without 3 µg/ml of antimycin A, and diluted so that the initial cell concentration for the Bioscreen C measurements was 10^5^ cells/ml. OD_600_ measurements were taken every 15 min for 7 days or until the cultures reached stationary phase. The same procedure was followed for the stationary growth experiments, replacing the HG medium from pregrowth and the final growth medium with 5% galactose.

The lag times were obtained by analyzing the growth curves with a homemade R script. Since the initial OD_600_ values are under the detection limit, the first three measurements were averaged to determine the background OD_600_ signal for each well. Once the background signal was subtracted, the nonlinearity between the optical density and the cell density was corrected using the following formula derived from reference 65:

$${OD}_{600,cx}={OD}_{600,me}+0.499{\left({OD}_{600,me}\right)}^{2}+0.191{\left({OD}_{600,me}\right)}^{3}$$

where the cx subscript stands for corrected and the me subscript stands for measured.

The growth rate was then obtained by calculating the discrete derivative of ln(OD_600,me_) versus time. The minimum growth rate (minR) was identified. In the case of two exponential phases in the growth curve (i.e., conditions with a mix of carbon sources in the medium), the maximum growth rate for each phase (maxR1, maxR2) was detected. Once these parameters were determined, the lag time was calculated by the time difference between two time points: the start and the end. The start of the lag is defined by the time corresponding to the intersection point between an exponential line touching the growth curve at the point corresponding to the maxR1 and a horizontal line crossing the point corresponding to the minR. The end of the lag is defined by the time corresponding to the intersection point between an exponential line touching the growth curve at the point corresponding to the maxR2 and a horizontal line crossing the point corresponding to the minR.

### Oxygen consumption rate measurement and analysis.

Cells were inoculated in a 96-well plate containing HG medium and serially diluted for growth overnight at 30°C shaking at 900 rpm. The next day, cultures at an OD_600_ of 0.1 were transferred to a new plate and serially diluted in fresh HG medium for growth overnight. The next day, 50-µl portions of cultures at an OD_600_ of 0.1 were inoculated in 100 µl of HG medium in a 96-well plate which has a fluorescent oxygen sensor embedded at the bottom of each well (OxoPlates; PreSens Precision Sensing). To calibrate the oxygen levels, air-saturated water and air-saturated HG medium were used as the 100% saturated condition, while the 0% saturated condition was measured by totally filling a well with water containing 10% Na_2_SO_3_ and covering the well with plastic seal. The OxoPlate with 5 biological replicates of each culture and all the calibration solutions was incubated for 24 h at 30°C in an automatic plate reader (Infinite M200 Pro; Tecan). Fluorescence intensity and OD_600_ were measured every 15 min for all wells.

To analyze the oxygen consumption rate during growth using the OxoPlates, a customized R script was used. To plot the growth curve, the background OD_600_ signal was subtracted from the OD_600_ measurements. Oxygen levels (oxygen partial pressure in percent air saturation) were calculated per time point using the calibration solutions, and the ratio of indicator phosphorescence to reference fluorescence of the optical sensor in the OxoPlate was determined. To plot the oxygen consumption rate, we corrected for the optical density of the cells by calculating the derivative of the smoothed oxygen level curve, divided by the corresponding optical density at that specific time point.

### Correlations between the lag time and the proteomics data of Skelly et al. (45).

The normalized peptide data from the supplemental information of the work of Skelly et al. (45) were downloaded. In case there was expression data for multiple peptides from a certain protein, all their expression data were averaged to obtain the final expression data of that protein. The lag times of 18 different natural S. cerevisiae strains were obtained using population growth measurements. A vector was constructed with the lag times of the 18 strains, from longer to shorter lag times. Similarly, and keeping the same order of strains, the expression data of one protein was introduced in a new vector. The Spearman correlation coefficient between both vectors was then calculated. This procedure was repeated for all the proteins in the downloaded data set.

### Single-cell time-lapse microscopy.

The setup used is similar to the one previously described (28) with some modifications. In brief, cells were pregrown on YP plus 5% galactose overnight. Cultures in exponential growth phase were transferred to fresh medium for growth overnight. The cultures at an OD_600_ of 0.1 were washed three times with HG medium, serially diluted, and incubated for 8 h at 30°C with shaking at 900 rpm. After that, the cultures at an OD_600_ of 0.1 were washed three times with YP plus 5% galactose, and 1 µl of each culture was spotted on an agar gel containing YP plus 5% galactose. This gel was positioned on the stage of an inverted automated Nikon Eclipse Ti fluorescence microscope with a 60× 1.4NA oil immersion objective. The microscope was located inside a temperature-controlled chamber set at 30°C. A Lambda XL lamp was used for illumination.

### Physiological characterization in bioreactors.

#### (i) Media and cultivation techniques.

The prototrophic haploid laboratory strain Saccharomyces cerevisiae strain CEN.PK113-7D (*MAT***a**) (66) and its *rip1*::*KanMX4* mutant IMK242 were used for cultivation in bioreactors (25, 67). Yeast cultures were grown in chemically defined medium (68) supplemented with 20 g/liter^−1^ glucose or with mixtures of glucose and galactose (10 g liter^−1^ each). These sugars were added to autoclaved media after separate heat sterilization of concentrated solutions at 110°C. Bioreactor batch cultivations were supplemented with 0.15 ml silicon antifoam (BDH, Poole, England). Anaerobic cultivations were also supplemented with the anaerobic growth factors Tween 80 (420 mg liter^−1^) and ergosterol (10 mg liter^−1^). To maximize experimental reproducibility, precultures for the bioreactor experiments were prepared according to the following strict protocol. A −80°C stock culture of CEN.PK113-7D was thawed and inoculated in 500-ml flasks containing 100 ml of chemically defined medium with 20 g liter^−1^ glucose and incubated at 30°C and 200 rpm. When the exponential phase was reached, a sample was transferred to a second set of two flasks containing 20 g liter^−1^ glucose chemically defined medium (initial biomass concentration of 0.25 g [dry weight] liter^−1^). After incubation at 30°C and 200 rpm, both precultures were harvested in early exponential phase and thoroughly mixed, washed once with sterile water, and inoculated into the bioreactors to give an initial biomass concentration of 25 mg (dry weight) liter^−1^. Aerobic and anaerobic batch cultivations on glucose-galactose mixtures were performed at 30°C in 2-liter laboratory bioreactors (Applikon, Delft, The Netherlands) with a working volume of 1.4 liters. The vessels were equipped with a rapid sampling port with capillary tubing to enable accurate internal metabolite analysis. Culture pH in the bioreactors was maintained at 5.0 by the automated addition of 2 M KOH. Cultures were stirred at 800 rpm and sparged with 0.7 liter min^−1^ air or nitrogen gas (<5 ppm O_2_; Linde Gas, Benelux). Norprene tubing was used to minimize oxygen diffusion into the bioreactors. Dissolved oxygen was monitored with an AppliSens oxygen electrode (Applikon).

#### (ii) Analysis of biomass, gas, and extracellular metabolites.

OD was measured at 660 nm in a Libra S11 spectrophotometer (Biocrom, Cambridge, UK). The bioreactor exhaust gas was cooled by a condenser connected to a cryostat set at 2°C and dried with a Perma Pure dryer (Inacom Instruments) before analysis of the O_2_ and CO_2_ concentrations with a Rosemount NGA 2000 analyzer. The gas flow rate was determined with an Ion Science Saga digital flow meter. Extracellular concentrations of ethanol, glucose, and galactose were measured in supernatants by HPLC analysis: a Bio-Rad Aminex HPX-87H column, eluted with 5 mM sulfuric acid at a flow rate of 0.6 ml min^−1^ and at 60°C was coupled to a Waters 2410 refractive index detector.

#### (iii) Analysis of intracellular adenosine-phosphate concentrations.

Samples for internal metabolite analysis were obtained by rapid sampling and were then quenched and processed as previously described (69). Intracellular AMP and ADP were determined enzymatically, using a previously described method (70). Intracellular ATP was assessed enzymatically as described elsewhere (71). The adenylate energy charge (EC) was calculated according to the following equation:

$$EC=\frac{\left(\left[ATP\right]+0.5\left[ADP\right]\right)}{\left(\left[ATP\right]+\left[ADP\right]+\left[AMP\right]\right)}$$

#### (iv) Enzyme activity assays.

Preparation of cell extracts and analysis of enzyme activities in the extracts were performed as described previously (25). Protein concentrations in cell extracts were determined by the Lowry method, using dried bovine serum albumin (fatty acid free; Sigma) as a standard.

#### (v) Transcript level analysis by quantitative PCR.

Expression levels of *GAL* genes were analyzed by quantitative RT-PCR using *ACT1* as the reference. Culture samples taken from bioreactors were immediately plunged into liquid nitrogen and stored at −80°C until further analysis. Total RNA extraction was performed as described previously (72). For each sample, the quality of total RNA was checked using Agilent RNA 6000 Nano reagents (Agilent Technologies Netherlands B.V., Amstelveen, The Netherlands) and the Agilent Bioanalyser 2100 according to the manufacturer’s instructions. First-strand cDNA synthesis was carried out using the QuantiTect reverse transcription kit (Qiagen Benelux B.V., Venlo, The Netherlands) with random primers and integrated removal of genomic DNA contamination. Quantitative real-time PCR was performed on a Qiagen Rotor-Gene Q instrument with the following settings: 95°C for 5 s, 60°C for 10 s, 72°C for 10 s, and plate reading. These steps were repeated for 40 cycles. A melting curve from 60 to 95°C was generated at the end of each reaction. The 20-µl reaction mixture consisted of 10 µl of Absolute Rotor-Gene SYBR Green I PCR kit (Qiagen), 1 µM forward primer, 1 µM reverse primer, and cDNA (10 or 1 ng µl^−1^). The primers used are listed in Data Set S1. Samples were taken from two independent batch cultivations, and each sample was analyzed in triplicate. The expression of each allele relative to *ACT1* was calculated as amplification*^ACT1^* ^take-off – sample take-off^.

#### (vi) Assessment of cell viability and metabolic activity.

Culture viability and metabolic activity were assayed by staining 10^7^ cells, freshly sampled from the bioreactors, with the *Funga*light CFDA, AM/propidium iodide yeast vitality kit (Invitrogen, Carlsbad, CA). The fluorescence of cells in the population was measured using a Cell Lab Quanta SC MPL flow cytometer (Beckman Coulter, Woerden, The Netherlands), following the procedure described in reference 73.
