# Supplementary material for: The Crabtree Effect Shapes the Saccharomyces cerevisiae Lag Phase during the Switch between Different Carbon Sources
Source: mBio. 2018 Oct 30;9(5):e01331-18. doi: 10.1128/mBio.01331-18 (PMC6212832; doi:10.1128/mBio.01331-18)
Supplement: FIG S1 [file mbo005184134sf1.pdf]

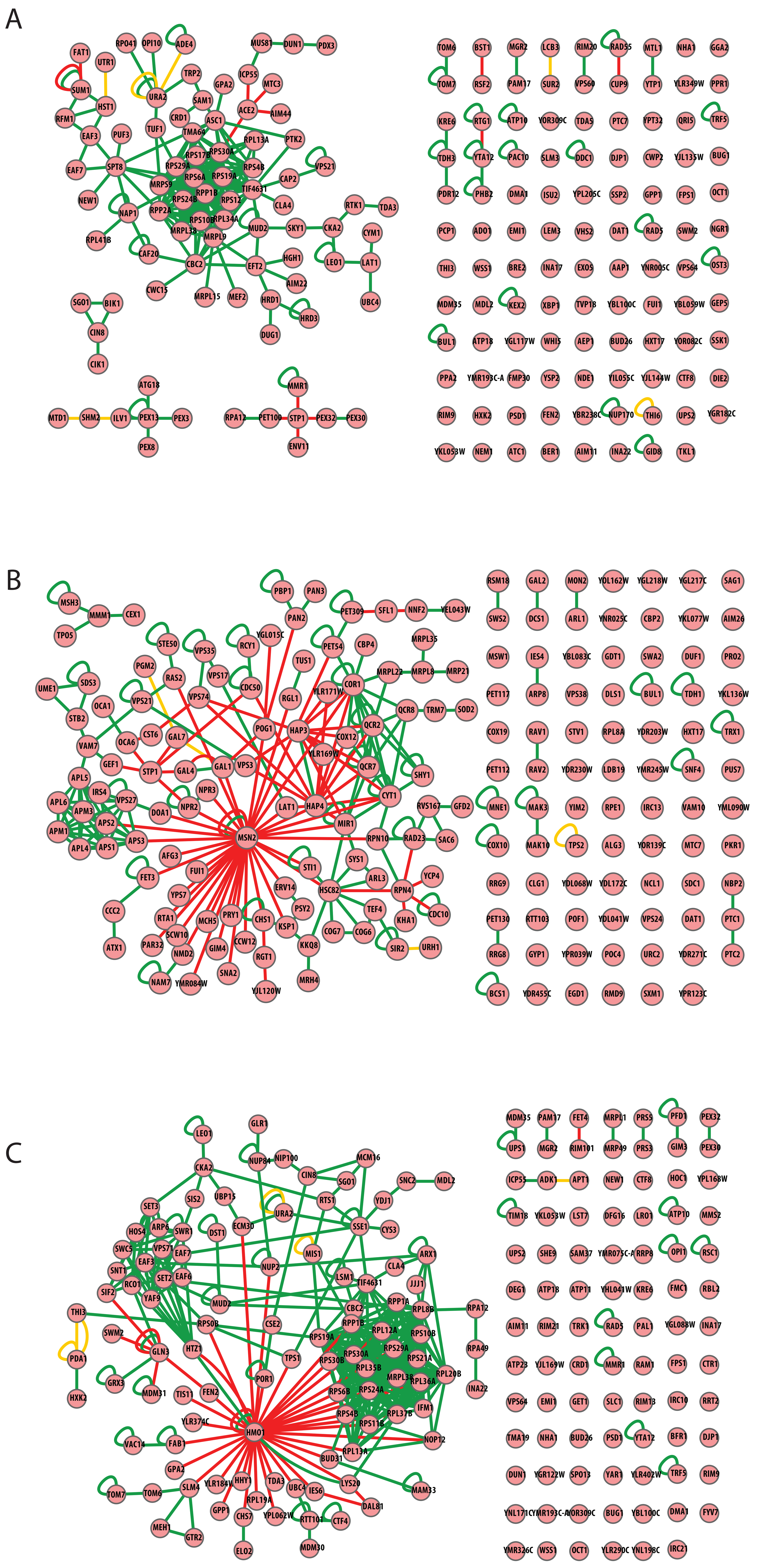

Fig S1: Interaction networks show the importance of different cellular processes in gradual and stable conditions. (A) Interaction networks of the 200 genes that when deleted show the strongest enrichment during glucose-to-galactose shift. (B) Interaction networks of the genes that, upon deletion show the strongest depletion when growing in galactose. (C) Interaction networks of the genes when deleted show the strongest enrichment when growing in galactose. In all networks, the edge colours correspond to protein-protein (green), methylation (yellow) and protein-DNA (red) interactions.

Fig S1
