# Supplementary material for: The Crabtree Effect Shapes the Saccharomyces cerevisiae Lag Phase during the Switch between Different Carbon Sources
Source: mBio. 2018 Oct 30;9(5):e01331-18. doi: 10.1128/mBio.01331-18 (PMC6212832; doi:10.1128/mBio.01331-18)
Supplement: FIG S2 [file mbo005184134sf2.pdf]

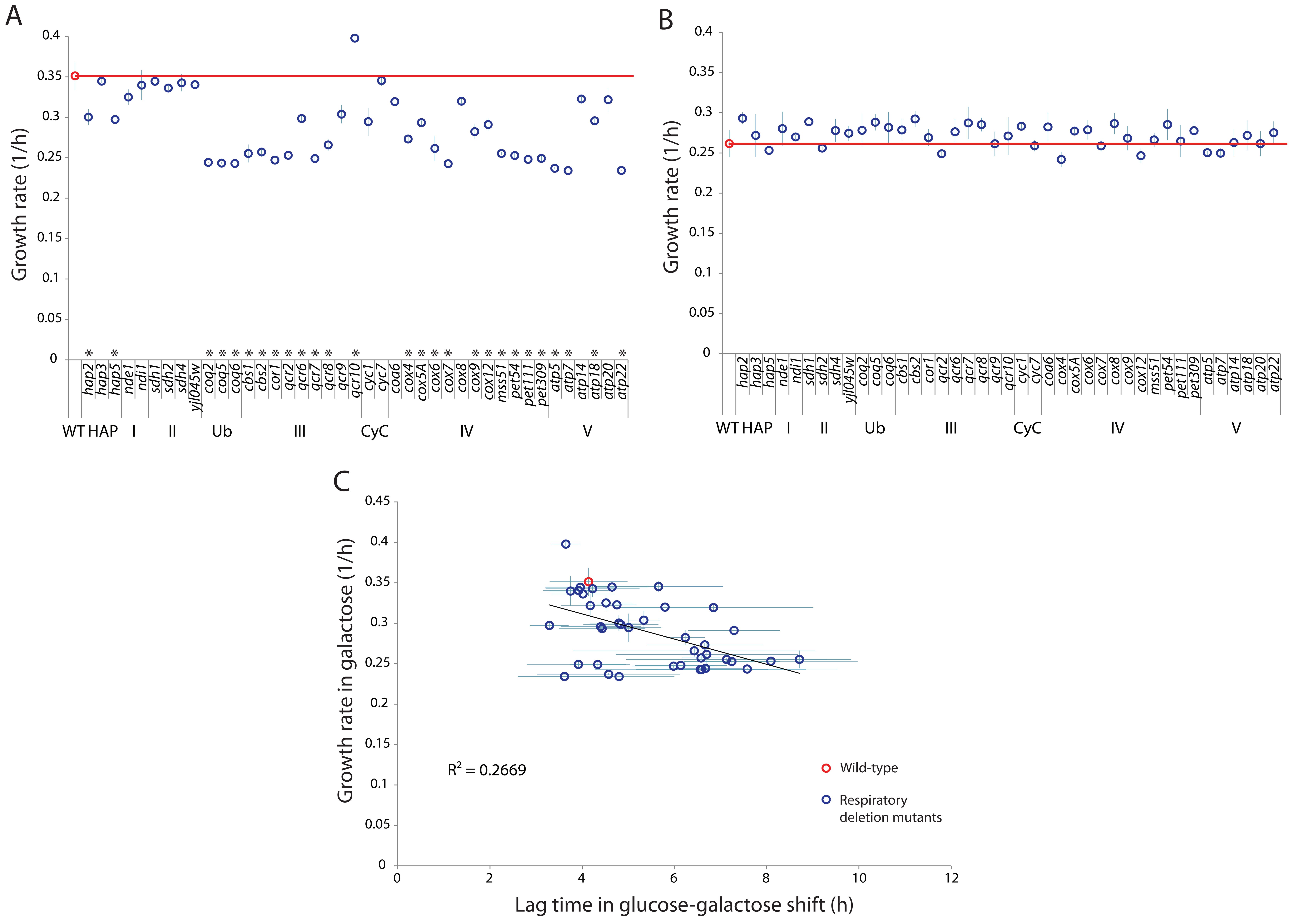

Fig S2: Respiration is needed for efficient growth on galactose. (A) Growth rates in galactose of mutants in which specific genes involved in respiration were deleted. The red circle and line indicate the growth rate of the wild-type. *p<0.05 (two-tailed Student's t-test). (B) The same as in A but medium supplemented with antimycin A. (C) Correlation between the growth rates of the respiratory deletion mutants when growing in galactose medium versus their lag times. The red circle indicates the wild-type. Error bars in all panels correspond to standard deviations of 3 or more biological replicates.
