# Supplementary material for: The Crabtree Effect Shapes the Saccharomyces cerevisiae Lag Phase during the Switch between Different Carbon Sources
Source: mBio. 2018 Oct 30;9(5):e01331-18. doi: 10.1128/mBio.01331-18 (PMC6212832; doi:10.1128/mBio.01331-18)
Supplement: FIG S3 [file mbo005184134sf3.pdf]

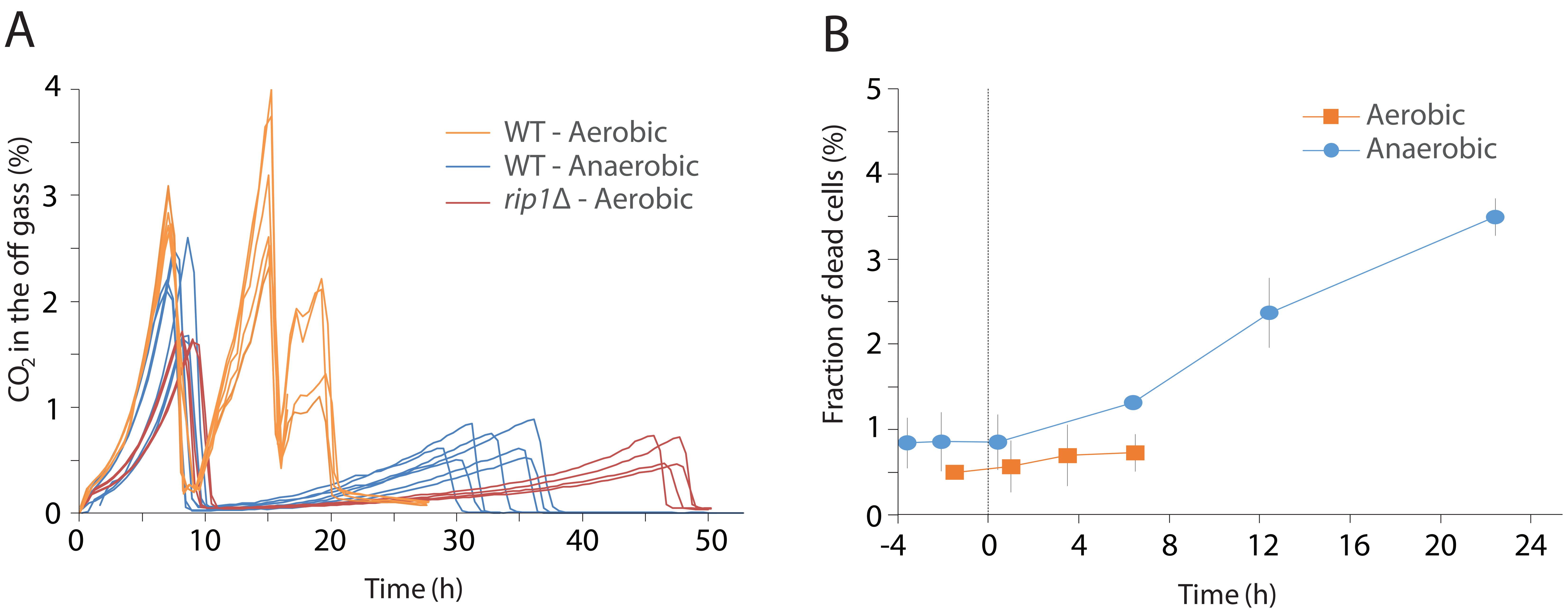

Fig S3: (A) CO_2 production of the CEN.PK prototrophic strain IMK242 carrying a *RIP1* deletion (van den Brink et al., 2009) when cultivated in aerobic bioreactors (as described in the Material and Methods) and compared to its isogenic reference strain *S. cerevisiae* CEN.PK113-7D during aerobic and anaerobic growth on glucose-galactose mixtures. For each culture condition at least four independent culture replicates are shown. (B) Percentage of dead cells (PI positive) was used to assess the viability of aerobic and anaerobic batch cultures. Error bars correspond to standard deviations of 2 biological replicates. The time of glucose depletion, corresponding to the shift to galactose consumption, is set as time zero and depicted as a dashed line.

Fig S3
